# The Formulation and Evaluation of *Melaleuca alternifolia* Cheel and *Cymbopogon flexuosus* Linn Essential Oils Emulgel for the Treatment of Vulvovaginal Candidiasis

**DOI:** 10.3390/gels9120949

**Published:** 2023-12-03

**Authors:** Adeola Tawakalitu Kola-Mustapha, Miracle Halima Aliu, Ronke Hadiyat Bello, Oluwakorede Joshua Adedeji, Yusuf Oluwagbenga Ghazali

**Affiliations:** 1 College of Pharmacy, Alfaisal University, Riyadh 11461, Saudi Arabia; 2 Department of Pharmaceutics and Industrial Pharmacy, Faculty of Pharmaceutical Sciences, University of Ilorin, P.M.B 1515, Ilorin 240101, Nigeria; 3 Department of Pharmaceutical Microbiology and Biotechnology, University of Ilorin, P.M.B 1515, Ilorin 240003, Nigeria

**Keywords:** *Melaleuca alternifolia*, *Cymbopogon flexuosus*, vulvovaginal candidiasis, emulgel

## Abstract

The global concern regarding the occurrence of antifungal resistance to synthetic conventional azoles used for treating vulvovaginal candidiasis, along with the associated side effects, is significant. Consequently, the pursuit for substitutes such as natural therapies has ensued. Essential oils, derived from plants, have been extensively researched and found to possess antibacterial and antifungal properties. This study aimed to assess the antifungal efficacy of two essential oils, both alone and in combination, against *Candida albicans*. Essential oils were formulated into an emulgel separately and as combinations. The essential oils of *Melaleuca alternifolia* and *Cymbopogon flexuosus* were used in this study. The resulting emulgel formulations were characterized for their antifungal activity against *Candida albicans*. Physiochemical properties such as pH, viscosity, and appearance were also determined. The prepared emulgels were thereafter observed for stability over a period of 1 month. The MIC of *Melaleuca alternifolia* was seen to be 50 µL/mL while *Cymbopogon flexuous* was seen to be more potent at 25 µL/mL against *C. albicans* exhibiting strong synergistic effect at 0.4. The emulgel formed was white in color, smooth on skin, and had the odor of the essential oils, which is sweet to the nose. The pH of the formulations with the essential oils were acidic in the range of 3.70–3.83, making them suitable for vagina application. The emulgels had viscosities ranging from 4417.6 to 8968.7 mPas, owing to the thickness of the essential oils contained. The emulgel formulation with the combination of essential oils was more potent that the two with individual essential oils; furthermore, the one with *Cymbopogon flexuous* was more potent than the one with *Melaleuca alternifolia*. Based on the properties of the formulated emulgels and their activity against the test organism, the preparations have significant potential in the management of vulvovaginal candidiasis.

## 1. Introduction

A dosage form is a formulation that combines an active pharmaceutical ingredient (API) with excipients to facilitate the process of dosing, administering, and delivering the drug to patients. Various dosage forms exist, which vary based on the route of administration and the location where the active components take effect. Dosage forms encompass a variety of options, including solids (such as tablets, capsules, powders, and granules), semisolids (such as creams, ointments, pastes, and gels), liquids (including syrups, elixirs, decoctions, and lotions), and gases (such as aerosols) [1]. The topical drug administration approach circumvents the first pass effect, a significant drawback of the oral route. However, its primary constraint lies in the difficulty of transferring hydrophobic chemical components through the skin [2].

Emulgels are an innovative medication delivery technology that combine the properties of both emulsions and gels. They are utilized for the delivery of various pharmaceuticals, such as analgesics, anti-inflammatory meds, anti-acne medications, and anti-fungal compounds. Emulgels have characteristics such as easy removal, spreadability, thixotropy, lack of greasiness, extended shelf life, and transparency [3]. Emulgels are gelled emulsions, which can be either water-in-oil or oil-in-water. This vehicle is highly suitable for hydrophobic medicines. Emulgels have the characteristics of both gels and emulsions, making them a dual-controlled release system capable of delivering both hydrophilic and lipophilic medicines. This is due to the presence of two distinct phases, namely aqueous and non-aqueous [4]. Emulgels are created by employing oil-in-water systems for lipophilic medications and water-in-oil systems for hydrophilic pharmaceuticals. An emulgel is formed when a gelling ingredient is added to a standard emulsion.

The vagina serves as a pathway for the administration of medications by topical application. Vaginal formulations are exclusively employed for the administration of locally acting medications, such as antimicrobials, spermicidal agents, antimycotics, hormone therapy, contraceptives, and others. The vaginal route is chosen because of the profusion of blood vessels compared to systemic preparations [5]. Drugs delivered vaginally can be absorbed through three primary mechanisms: transcellular, paracellular, and vesicular or receptor-mediated transport. The process of drug absorption from the vagina consists of two primary stages: drug breakdown in the vaginal lumen and penetration through the membrane [6].

Candidiasis is a prevalent opportunistic infection that can impact persons with weakened immune systems as well as those with normal immune function [7]. Candida species are a component of the typical human microbiota. However, if the immune system of the host is weakened or if other circumstances disturb the equilibrium of bacteria, candida can proliferate excessively and lead to illness. Candidiasis is a mycotic infection resulting from the overgrowth of the yeast known as candida. Candida, a type of yeast, is typically found on the skin, mucous membranes, and in the gastrointestinal tract. However, it can proliferate and lead to infection in specific circumstances [8]. Candidiasis can manifest in various forms, such as oral thrush, vaginal yeast infections, and invasive candidiasis. Vaginal yeast infections are a prevalent form of candidiasis, impacting around 75% of women at some stage in their lifetimes. Common symptoms include pruritus, stinging sensation, and discharge from the vaginal area [9]. Invasive candidiasis occurs when the yeast infiltrates the bloodstream, potentially causing grave sickness or fatality. Invasive candidiasis is more likely to occur in individuals who have recently undergone surgery, have been using antibiotics for an extended period, or are undergoing immunosuppressive medication [10]. Furthermore, candida can induce skin infections, including diaper rash, intertrigo, and nail infections [11]. Inadequately managed candidiasis can lead to systemic candidiasis, a severe type of infection that can develop in critically ill patients in intensive care units and which is linked to high fatality rates [12]. Vulvovaginal candidiasis (VVC) is characterized by inflammation of the vagina or vulva resulting from an infection caused by the candida yeast. It is the second most common cause of vaginitis, following bacterial vaginosis. The symptoms of VVC include pruritus in the vulva and vagina, white curd-like discharge, a sense of burning, erythema in the vulva and vagina, and dyspareunia [13]. According to reports, a minimum of 70% of women globally will experience at least one occurrence of VVC during their reproductive years [14].

Vulvovaginal candidiasis (VVC) is peculiar because of the difficulties encountered in its pharmacological management. The golden standard for treatment involves the use of fluconazole in affected women [15]. While this guideline of treatment has recorded notable successes over the years, recent trends in management have shown a pattern of resistance leading to inadequacy in therapeutic outcomes and reoccurrence of VVC after fluconazole therapy [16]. This worrisome development underscores the need for the development of novel alternatives for a more efficient management of VVC in women.

Different approaches have been deployed to improve the management of VVC and minimise tendencies for resistance in therapy. Some of these approaches have leveraged on the growing applications of nanotechnologies to enhance drug delivery. One such effort was the development of low-intensity ultrasound-mediated drug-loaded nanoparticles to facilitate the intravaginal delivery of amphotericin B against VVC [17]. Although this approach led to some improvement in the antifungal activity of the drug candidate (amphotericin B), the formulation technique (and required materials) can be characterized as quite sophisticated and may not be feasible for bulking-up and commercial development, especially in developing and underdeveloped regions of the world. As an improvement to nanotechnology-driven models in vulvovaginal drug delivery, interventions through phytomedicines are being sought. In line with this, Rusda et al. (2020) worked on the therapeutic effects of *Nigella sativa* plant extract on a female Wistar rats’ VVC model and obtained evidence of the relevance of plant derivatives as useful remedies in VVC [18]. Similarly, Córdoba et al. (2019) evaluated the activities of the essential oils of *Laurus nobilis*, *Thymus vulgaris*, *Mentha piperita*, *Cymbopogon citratus*, and *Lippia junelliana* against Candida species. In their submission, these essential oils showed significant levels of activity against the test fungi species [19]. Building on these exploits, this research seeks to develop a novel dosage form of two essential oils towards the improved management of VVC.

Essential oils are intensely concentrated substances obtained from various parts of plants, such as leaves, stems, flowers, seeds, roots, fruit rinds, resins, or barks. Plant essential oils consist of intricate combinations of natural chemicals, encompassing both polar and non-polar molecules [20]. They are a potential class of natural compounds that can suppress fungus. Terpenes and terpenoids included in essential oils can break the cell membranes of fungi, leading to their demise. The chitin structure present in the cell wall of fungus, which is absent in humans, makes it the primary focus for selectively harmful antifungal drugs [21]. The existence of bioactive substances in *Melaleuca alternifolia* has been scientifically proved to possess numerous advantageous qualities [22]. Due to its broad-spectrum anti-microbial characteristics, it has great potential for treating various illnesses. Similarly, *Cymbopogon flexuosus* exhibits efficacy against a wide range of pathogenic organisms. It has been employed for its antibacterial, antifungal, and antiviral properties [23]. Based on these possibilities, the objective of this study is to develop an emulgel containing *M. alternifolia* and *C. flexuosus* to treat vulvovaginal candidiasis caused by *Candida albicans.*

## 2. Results and Discussion

### 2.1. Physicochemical Analysis of the Essential Oils of Melaleuca alternifolia and Cymbopogon flexuosus

Table 1 shows the physicochemical characteristics of essential oils of *M. alternifolia* and *C. flexuosus*. Both selected essential oils were colorless to pale yellow in color, clear, mobile liquids with their characteristic smell and highly soluble in 20% *v*/*v* Tween-80.

### 2.2. Distribution of Vagina Candidiasis among Collected Samples

**Figure 1:**
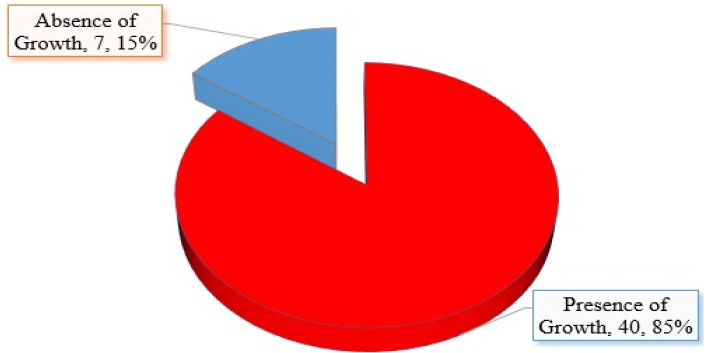
Prevalence of vagina candidiasis among collected samples.

Figure 1 shows the distribution of vagina candidiasis among samples collected. A total of 47 vagina swabs were collected and 40 (85.10%) vagina swabs yielded growth. The 40 vagina swabs that yielded growth were further identified based on the colonial morphology and Gram reactions as indicated in Table 2.

### 2.3. Antifungal Susceptibility Testing of Yeast Isolates

**Figure 2:**
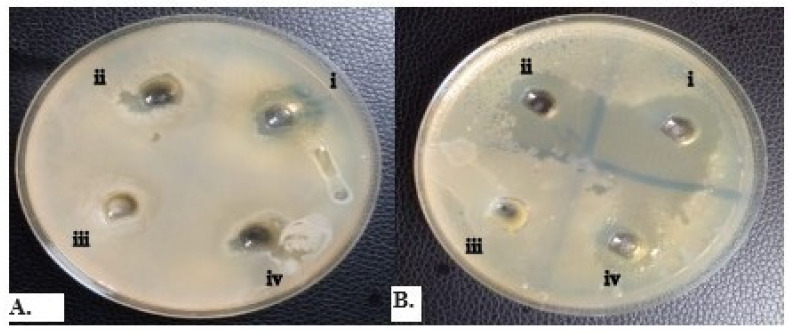
The anticandidal activity of *M. alternifolia* (**A**) and *C. flexuosus* (**B**) at (i) 100 µL/mL, (ii) 50 µL/mL, (iii) 25 µL/mL, (iv) positive control (nystatin) at 100 µL/mL; all measured by the diameter of the inhibition zone (mm).

Figure 2 shows the anticandidal activity of *M. alternifolia* and C. *flexuosus* as indicated by the clear zones of inhibition at 100 µL/mL to 25 µL/mL.

Table 3 shows the means of zones of inhibition of different concentrations of *M. alternifolia* and *C. flexuosus* against selected strains of *C. albicans*. Isolates CA28 exhibited the highest mean zones of inhibition of 21 ± 0.00 and 22.33 ± 0.58 mm at 100 µL/mL for both essential oils, while the least was observed against CA42 and CA46 at 8.16 ± 0.35 and 8.00 ± 0.00 for *M. alternifolia* and C. *flexuosus*, respectively. All *C. albicans* were sensitive to nystatin at 300 µL/mL.

### 2.4. Minimum Inhibitory Concentration (MIC) of Essential Oils

Table 4 shows the MIC of the *M. alternifolia* and *C. flexuosus* essential oils against *C. albicans*. The MIC ranges from 50 to 100 µL/mL and 25 to 100 µL/mL for *M. alternifolia* and *C. flexuosus* essential oils, respectively.

### 2.5. Synergistic Action of Melaleuca Alternifolia and Cymbopogon flexuosus Essential Oils in Combination

The results of the checkerboard bioassay at different mixing ratios are presented in Table 5 and Table 6. They shows the combined synergy of *C. flexuosus* and *M. alternifolia* at ratios 50:50 and 75:25, respectively, at a concentration range of 6.25–100 µL.

### 2.6. Post-Formulation Antifungal Activity

**Figure 3:**
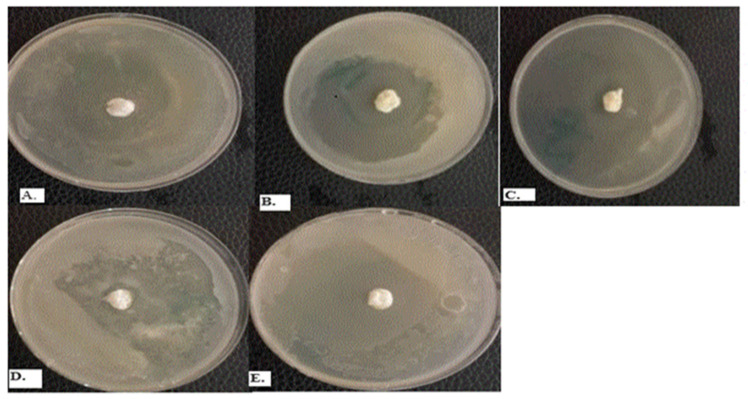
Antifungal activity of emulgel formulations as presented by zones of inhibition—(**A**) *Melaleuca alternifolia* emulgel; (**B**) *Cymbopogon flexuosus* emulgel; (**C**) combination of *Melaleuca alternifolia* and *Cymbopogon flexuosus* emulgel; (**D**) 1% clotrimazole emulgel (positive control); (**E**) emulgel only in DMSO (negative control).

Table 7 and Figure 3 show the zone of inhibition of the formulated emulgels against *Candida albicans* using 600, 900, and 1200 µL/mL. The highest inhibition (60 mm) was observed with *C. flexuosus* alone against *C. albicans* and in combination at 50 mm. Squatter colonies were observed with 500 mg/mL of 1% clotrimazole in DMSO with an approximate zone of inhibition of 20 mm.

### 2.7. Pseudo-Ternary Phase Diagram

**Figure 4:**
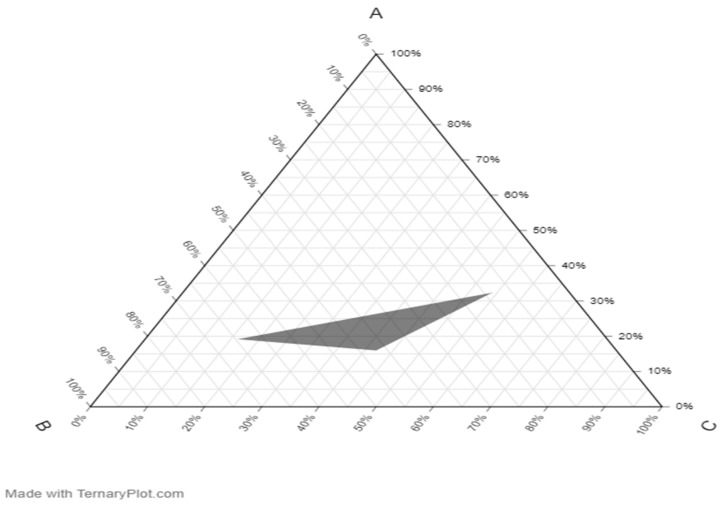
Pseudo-ternary plot of the emulsion prepared. A—Water; B—Oil; C—Smix.

The emulsion was optimized using the pseudo-ternary diagram. In this instance, a mixture of Smix (consisting of Tween 80 and Span 60) was included with coconut oil as the dispersed phase, while water served as the dispersing medium. The shaded area represents the region of stable oil-in-water emulsion within the constructed pseudo-ternary phase as presented in Figure 4. Each corner of the phase diagram represents 100% concentration of a constituent; A: water, B: oil, C: Smix.

### 2.8. Physicochemical Properties of Emulgel Preparation

Physicochemical properties of five emulgel preparations are presented in Table 8.

### 2.9. Stability Testing of the Formulations

The five emulgel formulations were evaluated for their stability after one week, two weeks, and one month and the results are presented in Table 9, Table 10 and Table 11.

### 2.10. Viscosity Evaluation of the Formulations

The viscosities of the different emulgel formulations are presented in Table 12.

Vulvovaginal candidiasis (VVC) is a prevalent health concern affecting women globally [24], including in Nigeria [25]. The 85% prevalence of *Candida albican* caused the VVC observed in our study is. However, prevalence higher than the 49.7% [26] and 32.6% [27] reported in Lahore—Pakistan and North central Nigeria among women, respectively. This shows varying prevalence globally and maybe due to factors such as socio-demographic factors, sample size, and changes in the pattern of antibiotic resistance. The problem of antibiotic resistance has prompted the investigation of natural remedies, specifically plants that contain essential oils such as coriander oil, lemon grass essential oil, eucalyptus oil, and others [28]. *Candida albicans* can switch morphology between yeast and filamentous growth, biofilm formation, and availability of enzymes such as lipases, phospholipases, and proteinase, thus increasing pathogenicity and the development of resistance [29]. Azole medicines and their derivatives are the preferred antifungal treatment for VVC. These treatments can be taken orally or used topically. Fluconazole, an azole, is losing its efficiency against clinical isolates of *C. albicans* despite its initial efficacy. As a result, there is a quest for alternative medicinal remedies for yeast infections that have high effectiveness and minimum negative consequences [30]. Compared to synthetic antimicrobials, natural compounds exhibit a wider range of activity, possess low toxicity, and have less adverse impacts on the environment [31], due to the integral role of plants in the ecosystem.

Emulgels, which are made by combining gel and emulsion, are a specific sort of topical drug delivery system [32]. They are utilized for administering various medications, such as antimicrobials, antifungals, antimycotics, and analgesics [4]. This study demonstrates the combined antifungal efficacy of two essential oils against vulvovaginal candidiasis, resulting in enhanced antifungal activity and reduced resistance. Previously, *Melaleuca alternifolia* has been investigated for its therapeutic applications, such as the treatment of VVC [33]. While *Cymbopogon flexuosus* has been studied for its antifungal properties in treating oral candidiasis, there is currently little research available on its usage for vulvovaginal candidiasis, despite claims of its antifungal activities [34]. This research enhances the presentation of essential oils from these plant species by producing an antifungal emulgel that is more appropriate for pharmaceutical applications, taking advantage of their therapeutic properties.

The essential oils assessment revealed antifungal activity against *C. albicans* with a minimum inhibitory concentration (MIC) of *M. alternifolia* was 50 µL, whereas the MIC of *C. flexuosus* was 25 µL. The findings indicate that both oils possess antifungal properties, as documented by Toungos et al. (2019), Silva et al. (2008), Shah et al. (2011), and Li et al. (2017) [34,35,36,37]. Both species exhibited strong efficacy against several strains of *Candida albicans*. *C. flexuosus* showed more efficacy in suppressing the growth of *C. albicans* compared to *Melaleuca alternifolia*. The MIC was determined as the amount of essential oil required (increased per gram) to create an emulgel that exhibits antifungal properties against *Candida albicans*. The use of a combination of interaction between essential oils of *M. alternifolia* and *C. flexuosus* is probably a new area to enhance treatment, improve antimicrobial efficacy, and reduce dosage exposure and human burden. The combined effect of the essential oils of *M. alternifolia* and *C. flexuosus* exhibited the strongest synergism with FICI values of 0.4 and 0.5 against both selected *C. albicans*. Synergism in activity between these oils has also been confirmed in some other studies [35,37].

The emulgels were created by utilizing coconut oil as the primary emulsion oil. The gel was produced by combining xanthan gum and guar gum in different ratios of 3:2, 2:3, 4:1, and 1:4 to determine the optimal gel formulation. The pH range considered normal for the vagina is 3.80 ± 0.20 [38]. The pH of the formulations including essential oils met the necessary pH range for vaginal use, rendering them compatible with the vaginal skin. The emulsion-only formulation had a pH of 3.16, while the emulgel without the essential oils had a pH range of 6.18–6.21. Consequently, it may be deduced that the essential oils decreased the pH of the emulgel system. The viscosity test conducted at a rotational speed of 60 revolutions per minute and a temperature of 30.4 °C yielded a viscosity of 4417.6 ± 8.2 mPas for the emulsion and 8694.1 ± 10.3 mPas for the unloaded emulgel. The emulgel, formulated with a blend of essential oils, exhibited a viscosity of 8946.3 ± 8.8 mPas at a temperature of 30.7 °C. These viscosities demonstrate how the thickness of the essential oils affects the internal friction of the final emulgel compositions. According to the reading, the viscosity of *M. alternifolia* essential oil is higher than that of *C*. *flexuosus* essential oil. Viscosity plays a crucial role in determining the extent to which an emulgel can spread; both temperature and storage time have an impact on it [39]. An emulgel by its design possesses dual qualities, incorporating the attributes of both an emulsion and a gel. When applied against *C. albicans* in this study, the formulated emulgel acted by diffusion from the point of application into the surrounding regions containing the applied Candida strains. By diffusing and releasing its component essential oil principles, the emulgel was able to inhibit the growth of selected *C. albicans* around it, leading to the generation of an inhibition zone as presented in Figure 3. However, in Figure 3D, the positive control using 500 mg/mL of 1% clotrimazole in DMSO exhibited squatter colonies, probably indicating a resistant mutant against tested agent. This has also been documented among bacteria such as uropathogenic *Escherichia coli* [40], probably suggesting a high level of misuse due to an easy route of administration and readily available nature.

Stability studies showed that the prepared emulgel maintained a consistent look and qualities for a duration of one month. This demonstrates the high compatibility between the essential oils and the emulgel base, resulting in negligible deterioration during the measured one-month timeframe. This information is documented in Table 9, Table 10 and Table 11. The results of the antifungal action demonstrate the heightened antifungal effect of the essential oils when used together, as opposed to when used separately. This is evident in the reduction in the required concentration to hinder the proliferation of organisms. Furthermore, this demonstrates that *C. flexuosus* exhibits greater potency in comparison to *M. alternifolia*.

The investigation conducted in this research study has been able to demonstrate the activity of the test essential oils and the plant-based emulgel formulated from them against *C. albicans;* the organism implicated in VVC in women. The experiments at this level have been in vitro, indicating that further in vivo assessment would be required to enable the translation of the outcomes of these formulations into clinical applications. The observed in vitro activity patterns of both essential oils and manufactured emulgels suggest that they could potentially be used to treat VVC caused by *C. albicans*.

The mechanism of antifungal activity of *C. flexuosus* has been reported to be associated with the presence of the phytochemicals: geranyl acetate, β-citronellol, citronellal, limonene, and citral in its essential oil [41]. These constituents act by depleting the plasma membrane of fungal organisms and inhibiting metabolic enzymes required for their functionality [41]. On the other hand, the antifungal activity of *M. alternifolia* has been linked with presence of terpinene-4-ol and 1,8-cineole in its essential oil [42]. These phytochemicals act on fungal organisms by inflicting damage on their cell membranes and other organelles, thereby leaving them vulnerable [42]. While these described mechanisms and the corresponding phytochemicals responsible for their action have been determined to be true for fungal organisms, the precise mechanism of action of the essential oils utilized against *C. albicans* in this study needs to be further studied.

Taking cues from the outcomes obtained in this project and in the spirit of widening drug delivery possibilities, synthetic antifungal agents like fluconazole and nystatin can be co-formulated in an emulgel base with the essential oils of *C. flexuosus* and *M. alternifolia* in future studies. Combining plant derivatives with an established record of activity with synthetic molecules for the prospects of synergy can be deployed as a way of combating resistance in antifungal agents. As illustrated by Endo et al. (2010) in their research where they combined the extracts obtained from pomegranate peels with fluconazole against *Candida albicans* to obtain some synergism in antifungal activity [43], this approach can be considered as a follow-up to this research project.

## 3. Conclusions

The results obtained from this study show the anti-fungal activities of both *M. alternifolia* and *C. flexuosus* against vaginal strains of *C. albicans* individually. The best antifungal activity was seen when both essential oils were combined at the ratio of 75:25 *C. flexuosus: M. alternifolia*. The essential oils of *C. flexuosus* and *M. alternifolia* in combination are relatively stable within the emulgel made of xanthan and guar gum, showing very minimal degradation over a period of one month. The formulated emulgel showed satisfactory physicochemical characteristics and suitability for use in the vagina. This combination, delivered as an emulgel, can be considered in the future for the clinical management of vulvovaginal candidiasis. If successfully developed, this emulgel has tendencies to be superior to currently available synthetic counterparts as it adopts the advantages of plant-based medicines in terms of its relative efficacy, safety, and the vast (natural) availability of the active therapeutic ingredients utilized in its formulation.

## 4. Materials and Methods

### 4.1. Materials, Equipment, and Isolates

Span 60 (J.T Baker Inc., Phillipsburg, NJ, USA), Tween 80 (J.T Baker Inc., Phillipsburg, NJ, USA), xanthan gum (Naturally Good Company, Maynila, Philippines), Guar gum (Naturally Good Company, Maynila, Philippines), Mueller–Hilton broth (MHB), Mueller–Hilton agar (MHA), Sabourad dextrose agar (SDA), brain heart infusion broth (BHIB) (Oxoid, Basingstoke, UK), coconut oil (Technical and Entrepreneurship Centre, University of Ilorin, Ilorin, Nigeria), resazurin dye (Canvax Biotech, Valladolid, Spain), normal saline (Juhel Nigeria Limited, Enugu, Nigeria), glycerol solution (Sigma-Aldrich^®^, Gillingham, UK), lemon grass essential oil (Piping rock^®^, Ronkonkoma, NY, USA), tea tree essential oil (Piping rock^®^, Ronkonkoma, NY, USA), distilled water (Central Research Laboratory, University of Ilorin, Ilorin, Nigeria), and Mycoten vaginal cream (DrugField^®^ batch no-22JO326, NAFDAC no-04-2809, Abuja, Nigeria). Electronic analytical weighing balance (Ohaus, Parsippany, NJ, USA), vortex mixer (XH-C1, M2 SCI, Allendale, NJ, USA), incubator (Microfield: model SM9092, 240 V and 2340 W, Dartmouth, UK), laminar air flow (Class II laminar flow Cellgard 220/240V, NuAire Inc. Plymouth, MN, USA), autoclave (PorTable 230 V, 1850 W, Cole-Parmer, Vernon Hills, IL, USA), refrigerator (LG: Model No. HRF-688-FF/A, Seoul, South Korea), and micropipettes (IndiaMART, Noida, India). *Candida albicans* (typed) (ATCC No. 10231) was obtained from the Laboratory Unit, Department of Microbiology and Biotechnology, Faculty of Pharmaceutical Science, University of Ilorin.

### 4.2. Ethical Considerations

Ethical clearance was obtained from the Ethical Review Committee of the State Ministry of Health (ERC/MOH/2023/06/120). In addition, oral consent was obtained from the women that met the study criteria.

### 4.3. Study Criteria (for Vaginal Swab Collection)

The inclusion criteria for this study included female patients who consented to participate in the study, registered in the selected health facility, and had symptoms suggesting vagina candidiasis as recommended by medical personnel. The exclusion criteria for this study included women on antibiotic therapy within 2 weeks before the study days and those who were not willing and not available at the time of study.

### 4.4. Physicochemical Analysis of Essential Oil

The physicochemical analysis of the essential oils of *M. alternifolia* and *C. flexuosus* was performed according to the protocol described by Zekri et al. (2023) [44]. The solubility properties of the essential oils were determined using varying concentration of Tween-80. All obtained parameters were noted and recorded appropriately.

### 4.5. Determination of Organoleptic Properties of the Essential Oils

This involved the analysis of the odor, color, and texture. All obtained parameters were recorded appropriately.

### 4.6. Microbiological Analysis

#### 4.6.1. Collection of Vaginal Swabs

A total of 47 high vagina swab (HVS) samples were collected with sterile cotton wool swabs according to the protocol described by Waikhom et al. (2020) [45]. Samples were collected by the licensed nurse after consent was obtained. Swab samples were carefully labeled and transported in BHIB medium in cold chain to the Laboratory Unit, Pharmaceutical Microbiology and Biotechnology Laboratory, University of Ilorin, for immediate analysis.

#### 4.6.2. Isolation and Identification of *Candida albicans*

Swabs were inoculated on prepared SDA plates and incubated aerobically at 37 °C for 24 h. Plates with no growth after 24 h were re-incubated for another 24 h to ensure their negativity, while those with colonial morphology on SDA plates were read and noted. Further identification was performed using Gram staining and germ tube test for large, budded yeast-like cells and filamentous extension of yeast cells after 3 h incubation in 500 µL human serum, respectively. All yeast isolates (40) were stored in BHIB with 25% glycerol at 4 °C for further analysis.

#### 4.6.3. Determination of Inoculum Size

Yeast inocula were prepared using the McFarland turbidity standard as described by Cheesbrough (2006) [46]. Freshly sub-cultured yeast isolates were inoculated into 2 mL normal saline and adjusted until their turbidity matched to 0.5 on the McFarland turbidity scale. This turbidity scale was prepared by adding 9.6 mL of 1% aqueous solution of barium chloride in 0.4 mL of 1% sulfuric acid, giving an approximate yeast density of 1.2 × 10^8^ CFU/mL.

### 4.7. Antifungal Assay

#### 4.7.1. Screening for Antifungal Activity of Essential Oils

The antimicrobial activity of individual essential oils were screened against selected yeast isolates using the agar well diffusion method as described by Katibi et al. (2022) [47]. Standardized inocula were seeded into prepared MH agar. Using sterile corkborer no 6 (6 mm in diameter), wells were made in the seeded agar plates and bottom sealed using molten agar. A volume of 100 µL of solubilized essential oils in 20% Tween-80 at concentrations of 100, 200, and 300 µL/mL were added into each well and allowed to pre-diffuse for 45 min. Incubation was thereafter performed at 37 °C for 24 h. The degree of antifungal activity was evaluated by measuring the zones of inhibition diameter in millimeters (mm). All tests were carried out in triplicate for each isolate.

#### 4.7.2. Determination of Minimum Inhibitory Concentrations (MIC) of the Essential Oils

The concentration of essential oils that yielded inhibitory effect in the agar well diffusion assay was used to determine the MIC using the microplate dilution method. Prepared MHB (100 μL) was placed in wells 2–9, then 200 μL of each of the essential oils solubilized in 20% Tween-80 was added to the wells 1. In well 2, 100 μL of 200 μL/mL essential oils was added to yield 200 μL/mL. From well 2, serial dilutions were performed up to well 9 and 100 μL removed from well 9 was discarded to yield a concentration range from 0.39 to 100 μL. Next, 50 μL of freshly prepared yeast turbidity was adjusted to approximately 0.5 McFarland turbidity standard to 1 × 10^8^ cfu/mL. Wells 10, 11, and 12 were set as broth, organism, and test-agent sterility control and microtiter plates were incubated at 37 °C for 24 h. Post incubation, 40 μL of resazurin dye was added to each well as an indicator of microbial growth. Plates were incubated at 37 °C for another 24 h. The lowest concentration, where there was no color change from blue to pink, was regarded as the MIC.

#### 4.7.3. Determination of Synergism of the Combination of the Essential Oils

This was performed using the broth microdilution checkerboard method as described by Satyajit et al. (2007) [44] with slight modification. A 96-well microtiter plate was used for this experiment. The plates were prepared under aseptic conditions. The MIC concentration was used as the stock solution. A volume of 100 µL of MHB was added into the rows from column 1–12. A volume of 100 µL each of the stock solutions of *M. alternifolia* essential oil was pipetted into row 1 from column 1–8. Then, a serial dilution was conducted in each column to give varying concentrations in a descending order. In column 8, 100 µL of stock solution of *C. flexuosus* essential oil was pipette into row 1–8. A serial dilution was conducted from column 8 down to column 1 to give varying concentrations and hence create a checkerboard sequence with varying combinations of both essential oils in each tube. A volume of 0.1 µL of 0.5 McFarland turbidity of the test organism was pipetted into each well from column 1–9. Columns 9–12 were the control tubes. Column 9 was for organism viability, column 10 for broth sterility, and columns 11 and 12 for essential oils sterility. The plates were incubated overnight at 37 °C and the MIC was read as the least dilution without any turbidity, as indicated after incubation with 40 μL of resazurin dye. A fractional inhibitory concentration index (FICI) was used to interpret the results [48]. Synergy is more likely to be expressed when the ratio of the concentration of each antibiotic to the MIC of that antibiotic is the same for all components of the mixture.

The ƩFICs were calculated as follows:

ƩFICs = FIC A + FIC B

where:

FIC A = The MIC of drug A in the combination/MIC of drug A alone.

FIC B = The MIC of drug B in the combination/MIC of drug B alone.

The combination is considered synergistic when the fractional inhibitory concentration (ƩFIC) index is ≤0.5. Indifference is indicated by an FIC index > 0.5 to ≤4, while antagonism is indicated when the ƩFIC is >4.

### 4.8. Pharmaceutical Analysis for Formulation of Emulgel

#### 4.8.1. Drug–Excipient Compatibility Study

The drug–excipient compatibility study was conducted by physically mixing the drug and excipients in a 1:1 ratio and subjecting them to 40 °C/75% RH for one month. Finally, Tween-80 was selected as the surfactant and Span 60 as the co-surfactant.

#### 4.8.2. Pseudo-Ternary Phase Diagrams for Emulsion

This was first conducted to obtain a formulation with a cloudy appearance, physical homogeneity, and easy flow ability. This was performed by varying the ratio of coconut oil as the oil phase to a mixture of Tween-80 as the surfactant and Span 60 as the co-surfactant to form a pseudo-ternary phase diagram. The surfactant and co-surfactant (Smix) were mixed at two different weight ratios (1:1 and 2:1). The coconut oil mixture and Smix were combined at different weight ratios from 1:9 to 9:1 in different glass vials for each phase diagram. The phase diagram was constructed using the template provided on ternaryplot.com.

#### 4.8.3. Determination of the Amount of Surfactant and co Surfactant for the Preparation of Emulsion Using the HLB System

Tween 80 (HLB of 15)

Span 60 (HLB of 4.7)

A = (100 (X − HLB A))/(HLB A − HLB B)

Tween 80 = 32%; Span 60 = 68%.

### 4.9. Formulation of the Essential Oil Emulgel

In a bottle, a measured quantity of coconut oil, the surfactant and co-surfactant, and water were mixed thoroughly by shaking vigorously as shown in Table 13. A weighed quantity of guar gum was mixed with a weighed quantity of xanthan gum and the mixture was dispersed into a measured quantity of water in a beaker to form a gel as described in Table 14. The mixture was thoroughly mixed and set aside to swell. The essential oils were blended into prepared emulsion; the essential oil-loaded emulsion was blended with the gel (1:1 ratio) of emulsion: gum. This was homogenized to generate an emulgel with the composition described in Table 15.

### 4.10. Characterization of the Emulgel

#### 4.10.1. Physical Appearance

The physical appearances of the formulated emulgels were observed visually and then examined for their organoleptic properties such as color; odor; consistency; and grittiness, which were evaluated by application on the skin.

#### 4.10.2. pH Measurement

The pH value was determined by using a standardized pH meter in a 1% aqueous solution of the sample formation.

#### 4.10.3. Viscosity

Each sample was poured into a container (beaker). Viscosity of each sample was taken using an NDJ-5S Digital Display Viscometer (Rinch Industrial Co. Limited, Shanghai, China) at 60 rpm with a spindle size of 4.

#### 4.10.4. Stability Test

The formulated emulgels were all subjected to stability studies to confirm their stability at room temperature (25 °C). Their appearance, color, spreadability and pH for each one was monitored after one week, two weeks, and one month post formulation [49].

#### 4.10.5. Determination of Homogeneity

The emulgels were all tested for homogeneity by visual inspection in their containers. They were all tested for presence of any aggregates.

### 4.11. Post-Formulation Antifungal Analysis of Emulgels

The solutions of the formulated emulgels were prepared with dimethyl sulfoxide as the solvent. The antifungal activities of the emulgels were tested using the agar well diffusion method as described by Katibi et al. (2022) [47]. A standardized inocula was used to flood plates of agar that had been prepared and labeled. On the inoculated plates, central holes were bored using a sterile corkborer no. 6. Then, the base of the holes was sealed using a few drops of molten agar. The emulgel solutions were then filled into the cylindrical holes. The prepared plates were then allowed to sit for a while for adequate diffusion. The plates were then incubated at 37 °C for 24 h. The zones of inhibition observed were measured along the diameter of the wells. All tests were carried out in triplicate for each isolate.

## Figures and Tables

**Table 1:** Physicochemical analysis of the selected essential oils (EO).

EO	Color	Clarity	Form	Odor	Solubility	Density (g/mL)
*M. alternifolia*	Colorless	Clear	Flowing Liquid	Fresh Lemony	+	0.898
*C. flexuosus*	Pale yellow	Clear	Flowing Liquid	Fresh-mint	+	0.984

+ = solubility in 20% Tween-80.

**Table 2:** Identification of yeast isolates.

S/No	Identification Criteria	Characteristics
1	Colonial Morphology	White to cream colored, smooth, yeast-like appearance, and pasty concave colonies
2	Gram Reaction	Gram positive, budded large oval cells

**Table 3:** Antifungal activity of essential oils against selected *Candida albicans* isolates.

Mean Zone of Inhibition (mm)						
Isolate Code	Concentrations of Essential Oils (µL/mL)					
	*Melaleuca alternifolia*			*Cymbopogon flexuosus*		
	100	200	300	100	200	300
CA01	12.00 ± 0.00	+	+	14.76 ± 0.70	+	+
CA02	16.83 ± 0.76	+	+	17.50 ± 0.35	+	+
CA14	14.16 ± 0.00	+	+	17.67 ± 0.70	+	+
CA17	14.67 ± 0.70	+	+	18.00 ± 0.00	+	+
CA23	18.17 ± 0.29	+	+	20.00 ± 0.00	+	+
CA28	21.00 ± 0.00	+	+	22.33 ± 0.58	+	+
CA34	16.83 ± 0.76	+	+	20.17 ± 0.29	+	+
CA39	10.00 ± 0.00	+	+	12.83 ± 0.76	+	+
CA42	8.16 ± 0.35	+	+	9.67 ± 0.58	+	+
CA46	8.33 ± 0.70	+	+	8.00 ± 0.00	+	+
Typed	20.00 ± 0.00	+	+	21.16 ± 0.29	+	+

Mean ± SD, *n* = 3 keys: typed = *Candida albicans* 10231; + = inhibition; nystatin at 300 µL/mL (positive control) = +.

**Table 4:** The MIC of essential oils against *C. albicans*.

Code	MIC (µL/mL)	
	*M. alternifolia*	*C. flexuosus*
CA01	100	100
CA02	50	25
CA14	100	50
CA17	100	100
CA23	50	50
CA28	100	100
CA34	100	100
CA39	No inhibition	100
CA42	No inhibition	100
CA46	No inhibition	No inhibition
Typed	50	25
*C. albicans* (Typed) = ATCC 10231		

**Table 5:** The checkerboard bioassay against *Candida albicans* at the ratio of 50:50.

Concentration (µL)	1 100	2 50	3 25	4 12.5	5 6.25	6 Organism Viability	7 Essential Oil Sterility	8 Broth Sterility
A	+	+	+	-	+	+	-	-
B	+	+	+	+	+	+	-	-
C	+	+	+	+	+	+	-	-
D	+	+	+	+	+	+	-	-
E	+	+	+	-	-	+	-	-
F	+	+	+	+	+	+	-	-
G	+	+	+	-	+	+	-	-
H Typed	+	+	+	+	+	+	-	-

Presence of growth = +; absence of the growth = -. FIC A = 0.125, FIC B = 0.25, ƩFIC = 0.4 (synergistic).

**Table 6:** The checkerboard bioassay against *Candida albicans* at the ratio of 75:25 *Cymbopogon flexuosus: Melaleuca alternifolia*.

Concentration (µL/mL)	1 100	2 50	3 25	4 12.5	5 6.25	6 Organism Viability	7 Essential Oil Sterility	8 Broth Sterility
A	-	+	+	+	+	+	-	-
B	-	+	+	+	-	+	-	-
C	-	+	+	+	+	+	-	-
D	-	-	+	+	+	+	-	-
E	-	-	+	+	+	+	-	-
F	-	+	+	+	-	+	-	-
G	-	+	+	+	-	+	-	-
H Typed	-	-	+	+	-	+	-	-

Presence of growth = +; absence of the growth = -; FIC A = 0.125, FIC B = 0.25, ƩFIC = 0.4 (synergistic).

**Table 7:** Antifungal activity of emulgel formulations.

Zones of Inhibition (mm)			
Essential Oils	600 µL/mL	900 µL/mL	1200 µL/mL
*Melaleuca alternifolia*	35	33.75	28.75
*Cymbopogon flexuosus*	38	14.75	60
Combination of the two essential oils	6.5	22.50	50
Emulgel only in DMSO	0.0	0.0	0.0

The zone of inhibition of 1% clotrimazole (positive control) was ~20 mm.

**Table 8:** Physicochemical properties of the emulgel preparations.

Formulations	F1	F2	F3	F4	F5
Color	White	White	White	Faintly yellow	Faintly yellow
Texture	Smooth	Smooth	Smooth	Smooth	Smooth
Odor	Coconut smell	Coconut smell	Tea tree smell	Lemon grass smell	Lemon grass smell
pH	3.16	6.18–6.21	3.70–3.74	3.81–3.83	3.76–3.78
Homogeneity	Good	Good	Good	Good	Very Good

**Table 9:** Physical evaluation after one week at 25 ± 5 °C.

Formulation	F1	F2	F3	F4	F5
Appearance	Smooth	Smooth	Smooth	Smooth	Smooth
Color	White	White	White	Faintly yellow	Faintly yellow
Odor	Coconut smell	Coconut smell	Tea tree smell	Lemon grass smell	Lemon grass smell
Homogeneity	Good	Good	Good	Good	Good
Texture	Smooth	Smooth	Smooth	Smooth	Smooth

**Table 10:** Physical evaluation after two weeks at 25 ± 5 °C.

Formulation	F1	F2	F3	F4	F5
Appearance	Smooth	Smooth	Smooth	Smooth	Smooth
Color	White	White	White	Faintly yellow	Faintly yellow
Odor	Coconut smell	Coconut smell	Tea tree smell	Lemon grass smell	Lemon grass smell
Homogeneity	Good	Good	Good	Good	Good
Texture	Smooth	Smooth	Smooth	Smooth	Smooth

**Table 11:** Physical evaluation after one month at 25 ± 5 °C.

Formulation	F1	F2	F3	F4	F5
Appearance	Smooth	Smooth	Smooth	Smooth	Smooth
Color	White	White	White	Faintly yellow	Faintly yellow
Odor	Coconut smell	Coconut smell	Tea tree smell	Lemon grass smell	Lemon grass smell
Homogeneity	Good	Good	Good	Good	Good
Texture	Smooth	Smooth	Smooth	Smooth	Smooth

**Table 12:** Viscosity measurements of the emulgel formulations.

Formulation	Torque	Temperature (°C)	RPM	Spindle No	Reading mPas
F1	44.2	30.4	60	4	4417.6 ± 8.2
F2	86.9	30.4	60	4	8694.1 ± 10.3
F3	89.9	30.6	60	4	8968.7 ± 7.8
F4	87.3	30.6	60	4	8734.9 ± 9.7
F5	89.4	30.7	60	4	8946.3 ± 8.8

**Table 13:** Composition of emulsion preparation.

Ingredients	A	B	C	D	F	G
Tween 80 (g)	2.56	1.60	0.64	2.88	2.24	1.92
Span 60 (g)	5.44	3.40	1.36	6.12	4.76	4.08
Coconut Oil (g)	2.00	5.00	8.00	1.00	3.00	4.00
Water (g)	4.78	1.90	2.37	13.09	12.17	8.18

**Table 14:** Composition of gel preparation.

Ingredients	A	B	C	D
Xanthan gum (g)	2.00	3.00	4.00	1.00
Guar gum (g)	3.00	2.00	1.00	4.00
Water (g)	45.00	45.00	45.00	45.00

**Table 15:** Composition of emulgel formulation.

Ingredients	F1	F2	F3	F4	F5
Emulsion (g)	3.00	1.50	1.50	1.50	1.50
Gel (g)	0.00	1.50	1.50	1.50	1.50
*Melaleuca alternifolia* essential oil (µL)	0.00	0.00	1500	0.00	300
*Cymbopogon flexuosus* essential oil (µL)	0.00	0.00	0.00	1500	1200

## Data Availability

The data presented in this study are openly available in the article.
